# Achievement of three year remission in a case of aggressive glioblastoma using a multidisciplinary treatment strategy: A case report

**DOI:** 10.3892/ol.2014.1937

**Published:** 2014-03-05

**Authors:** ATSUSHI ISHIDA, MIKA WATANABE, SEIGO MATSUO, KAKU NIIMURA, HARUKO YOSHIMOTO, KEIZOH ASAKUNO, HIDEKI SHIRAMIZU, AKIO NEMOTO, MIKI YUZAWA, TOMOKATSU HORI

**Affiliations:** 1 Department of Neurosurgery, Moriyama Memorial Hospital, Tokyo 134-8608, Japan; 2 Department of Pathology, Tohoku University Graduate School of Medicine, Sendai, Miyagi 980-8575, Japan

**Keywords:** glioblastoma, temozolomide, CyberKnife, bevacizumab, autologous formalin-fixed tumor vaccine

## Abstract

Glioblastoma (GB) is the most common type of malignant tumor of the central nervous system and, despite extensive research, its prognosis is poor. Although recent advances have been made in the treatment of GB with aggressive resection combined with radiochemotherapy, more than three-quarters of GB patients succumb to the disease within two years. The current study presents a highly aggressive case of small cell GB as diagnosed by histological features and immunohistochemistry for vimentin, glial fibrillary acidic protein, oligodendrocyte lineage transcription factor 2, isocitrate dehydrogenase 1-R132H and p53. The patient was treated using a multidisciplinary treatment strategy, which included temozolomide, CyberKnife radiotherapy and autologous formalin-fixed tumor vaccination. In addition, the patient developed radiation necrosis, which was treated with bevacizumab. In conclusion, three years following the initial diagnosis, the patient continues to experience a successful clinical course, and the observations of the current study demonstrate that a multidisciplinary treatment strategy may be effective for the treatment of aggressive GB.

## Introduction

Despite contemporary surgery, image-guided radiotherapy and chemotherapy, glioblastoma (GB) persists or relapses in almost all patients, with tumors almost always recurring locally (1). The management of recurrent GB is variable, but approaches include the best supportive care, second surgery, reirradiation and/or systemic therapy. Promising novel therapies for GB include temozolomide (TMZ) (2), stereotactic radiotherapy [such as Gamma Knife (3) and CyberKnife (4)], immunotherapy (5) and antiangiogenic agents, including bevacizumab [a monoclonal antibody against vascular endothelial growth factor (VEGF)] (6). Emerging data suggests that the use of these therapies alone or in combination may be safe and effective (7–10).

The current study presents a case of highly aggressive GB treated with TMZ, CyberKnife radiotherapy and concurrent autologous formalin-fixed tumor vaccination (AFTV; a novel tumor vaccine consisting of autologous formalin-fixed tumor fragments) (11). Following two years without recurrence, the patient’s condition deteriorated due to radiation necrosis, which subsequently lead to the initiation of a bevacizumab infusion, as previous studies have suggested that the treatment may reduce tumor necrosis (12–15). The patient’s condition and magnetic resonance imaging (MRI) results markedly improved and, thus far, the patient has remained well and without recurrence. Patient provided written informed consent.

## Case report

**Figure 1:**
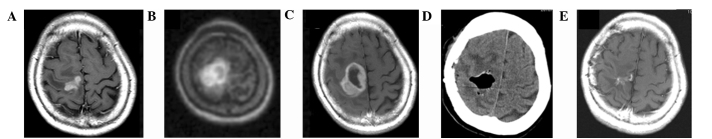
Imaging studies of the brain tumor at various stages of treatment. (A) Gadolinium-enhanced MRI captured during the patient’s initial visit showed evidence of an enhanced lesion near the right central sulcus and (B) ^11^C-methionine positron emission tomography revealed a lesion hot spot. (C) Gadolinium-enhanced MRI showed marked growth of the lesion in less than one month. (D) Computed tomography image of the cavity following tumor removal. (E) Gadolinium-enhanced MRI showed no recurrence approximately two years following surgery. MRI, magnetic resonance imaging.

A 58-year-old female presented with left leg seizures to the local hospital. The patient had been well prior to admission. On physical examination, the patient’s vital signs were normal, as were the results of the laboratory tests. Gadolinium-enhanced brain MRI revealed a mass (2-cm in diameter) around the right central sulcus (Fig. 1A). Due to a suspected high-grade glioma, the patient was transferred to the Moriyama Memorial Hospital (Tokyo, Japan) for further diagnosis and treatment. As ^11^C-methionine positron emission tomography (MET PET) is useful for evaluating grade, type and proliferative activity of astrocytic tumors (16), the patient underwent MET PET, which showed a ‘hot’ lesion (Fig. 1B) and lead to the suspicion of a malignant glioma. The seizures were intractable and, therefore, antiepileptic drugs were administered. In addition, a progressive left hemiparesis was observed. The tumor was highly aggressive and showed rapid growth in less than one month, as monitored by MRI (Fig. 1C).

The tumor removal by craniotomy was immediately performed under motor-evoked potential monitoring (MEP). A parietal midline craniotomy was carried out and the right central sulcus was identified by N20 phase reversal using sensory-evoked potential. The corticotomy was performed just behind the sulcus, during which gray glioma-like tissues were removed and submitted for pathological analysis. Subtotal removal of the tumor was accomplished without any MEP abnormalities (Fig. 1D). Additional treatment with TMZ, CyberKnife radiotherapy and AFTV was also initiated following surgery, and the patient’s condition remained stable without recurrence for approximately two years (Fig. 1E).

**Figure 2:**
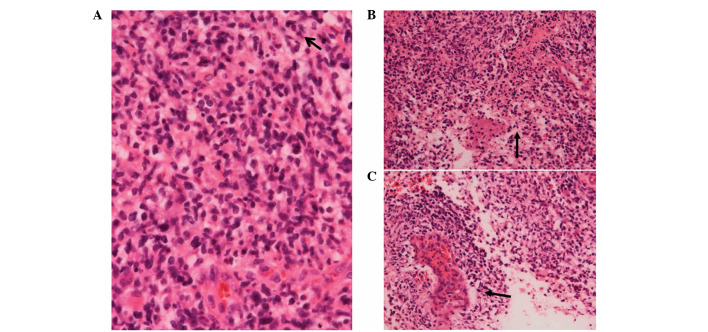
Microscopy observations. The tumor consisted of (A) monotonous, small atypical astroglial cells (magnification, ×200) and (B) necrosis with a pseudopalisading arrangement of neoplastic cells as indicated by the arrows (magnification, ×100). (C) Glomeruloid microvascular proliferation was observed as indicated by arrows, consistent with the diagnosis of small cell glioblastoma (magnification, ×100).

**Figure 3:**
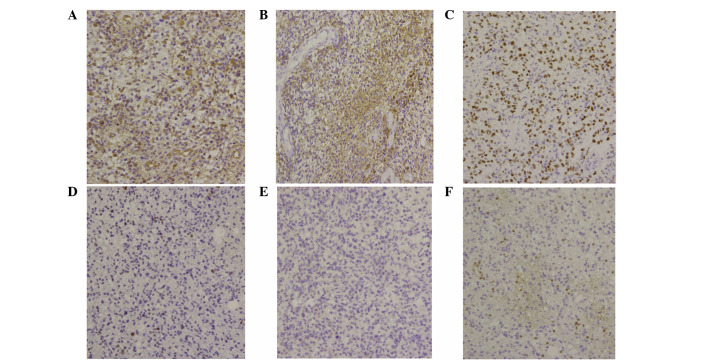
Immunohistochemistry: The glioma stained positive for the astrocytic markers, (A) vimentin, (B) glial fibrillary acidic protein and (C) oligodendrocyte lineage transcription factor 2 and negative for (D) p53 and (E) isocitrate dehydrogenase-R132H. (F) Immunoreactivity for O6-methylguanine DNA methyltransferase was weak (score of 1+) (magnification, ×200).

The histopathological analysis of the specimen revealed that the tumor consisted of atypical glial cells with a high nuclear to cytoplasmic ratio, proliferating in a fine fibrillary background (Fig. 2A). A stream-like arrangement of the spindled neoplastic cells, as well as a perivascular pseudorosette-like aggregation were also detected (Fig. 2B). These histological features indicated astrocytic characteristics. The neoplastic cells were also small and relatively homogeneous with only mild pleomorphism, although, the atypia of the neoplastic cells was high. The mitotic figures (5–6 mitoses/10 high-power fields), glomeruloid or epithelioid microvascular proliferation (Fig. 2C) and pseudopalisades were also detected. On the basis of these histological features, a diagnosis of small cell GB, World Health Organization (WHO) grade IV (17) and St. Anne-Mayo grade IV (18), was determined. In addition, the immunohistochemistry results were consistent with the diagnosis of GB, revealing strong immunoreactivity for vimentin (Fig. 3A) and moderate positivity for glial fibrillary acidic protein (Fig. 3B) and oligodendrocyte lineage transcription factor 2 (Fig. 3C). However, secondary GB could not be confirmed, as the immunohistochemistry results for p53 and isocitrate dehydrogenase 1 (IDH1)-R132H were negative (Fig. 3D–E) and the O6-methylguanine-DNA methyltransferase staining was weak with only <20% of positive cells (score of 1+; Fig. 3F).

The clinical and pathological observations indicated a highly aggressive case of GB and, therefore, additional therapies were required. The patient received CyberKnife radiotherapy (30 Gy in five fractions for five consecutive days) with TMZ (75 mg/m^2^/day for 42 consecutive days). The patient was also administered three courses of AFTV treatment, which was prepared as previously described (19), with no adverse events. The patient continued TMZ treatment at 100 mg/m^2^/day for five days every 28 days; however, the patient’s lymphocyte count began to decline and subsequently, the TMZ treatment was discontinued.

**Figure 4:**
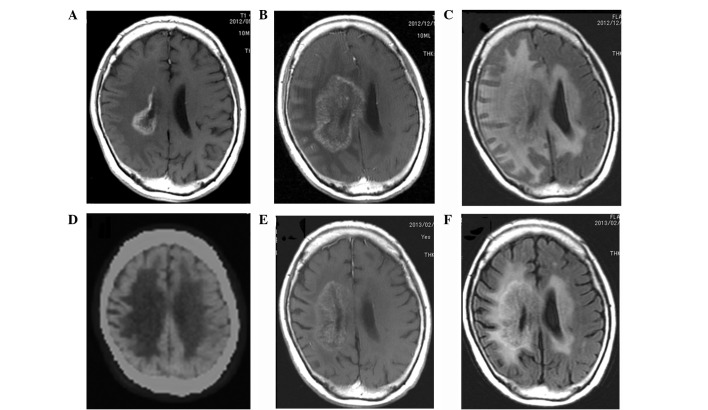
Imaging studies of the patient’s radiation necrosis. (A) Gadolinium-enhanced MRI showed an enhanced lesion caudal to the original lesion that appeared approximately two years following the initial surgery. (B) Gadolinium-enhanced MRI and (C) FLAIR showed growth in the size of the lesion following the second round of CyberKnife radiotherapy. (D) ^11^C-methionine positron emission tomography showed that the lesion and its surrounding area exhibited no hot spots, which is consistent with the diagnosis of radiation necrosis rather than tumor recurrence. Bevacizumab treatment showed a marked effect on the radiation necrosis lesion as visualized by gadolinium-enhanced (E) MRI and (F) FLAIR. MRI, magnetic resonance imaging; FLAIR, fluid attenuated inversion recovery.

Initially, the patient remained well without TMZ treatment; however, three months later, the patient was transferred to our hospital due to seizures and aggravation of the left hemiparesis. The MRI studies performed on admission showed an enhanced lesion caudal to the original lesion, which was considered to be a recurrence (Fig. 4A). Subsequently, the patient underwent a second cycle of CyberKnife radiotherapy, as it was considered to be the best treatment option at the time. Although the radiotherapy was administered without any adverse events, following the treatment, the patient’s symptoms appeared to worsen. Eventually, the patient’s condition declined to the point where the patient was unable to move unaided and, therefore, was readmitted to our hospital. Gadolinium-enhanced brain MRI on admission revealed an increase in the lesion size and fluid-attenuated inversion recovery image showed outstanding perifocal edema (Fig. 4B). This lead to the suspicion that the lesion was not due to tumor recurrence, but rather radiation necrosis. MET PET was performed and, similar to the gadolinium-enhanced brain MRI, no hot spot was detected (Fig. 4C). These results supported the diagnosis that the lesion was radiation necrosis. Several reports have suggested that bevacizumab is an effective treatment for radiation necrosis (12–15) and, therefore, the patient was enthusiastic to receive this treatment option. Bevacizumab was administered at a dose of 5 mg/kg every two weeks and, although the patient became hyperactive immediately following bevacizumab treatment, no adverse events were noted. Following three courses of bevacizumab, the MRI revealed a marked effect (Fig. 4D), which lead to the administration of three additional cycles of bevacizumab. Following meticulous rehabilitation, the patient’s condition continued to improve and, finally, with family support, the patient was discharged and returned home.

## Discussion

The current study presents a case of highly aggressive GB with a hot spot as visualized by MET PET, highly mitotic figures as revealed by pathological study and rapid growth as evaluated by MRI. A multidisciplinary treatment strategy was used and, three years following treatment, the patient remains well without recurrence. However, at one point, the patient’s symptoms did become aggravated due to radiation necrosis, which was successfully treated using bevacizumab. The standard treatment for GB is stereotaxic radiotherapy with TMZ and, in the present study, subtotal removal of the tumor was initially performed, which was followed by the immediate initiation of CyberKnife radiotherapy with TMZ. AFTV using paraffin-embedded tissues was also administered with the predicted outcome of additional antitumor activity.

Small cell GB is a recognized subtype of GB with a highly aggressive biology, which is classified as grade IV according to the WHO grading system. These tumors generally arise in the cerebral hemispheres of adults (20,21) and do not normally appear different from ordinary GB. However, microscopy may reveal features of small cell GB morphology, including the uniform size of cells with minimal pleomorphism and monomorphic round to oval nuclei. As with ordinary GB, microvascular proliferation and pseudopalisading necrosis may also be detected. In the present study, a number of mitotic figures and apoptotic cells were also observed, supporting a high proliferative activity (20,21). The IDH1-R132H mutation is a key factor in the biology and prognosis of gliomas, and it has been shown that patients with GBs with the IDH-1-R132H mutation exhibit improved outcomes compared with patients with wild-type IDH1 (22). It has also been shown that the IDH-1 mutation is likely to occur during the earlier stages of glioma tumorigenesis; therefore, a large proportion of low-grade gliomas possess the IDH-1 mutation. In addition, IDH1-R132H mutant-type GB may be indicative of a secondary GB progressing from low-grade glioma (23). In the present case, the immunohistochemistry results for the IDH1-R132H mutation were negative, suggesting that the primary small cell GB carried the wild-type IDH-1. The negative staining for p53 (Fig. 3D) also supported the diagnosis of primary GB; however, the differences in molecular abnormalities between small cell and ordinary GB remain undefined. The results of the current morphological analysis suggest a more aggressive phenotype for small GB than ordinary GB, but the prognostic significance of this morphological observation requires further investigation.

There has been a growing interest in therapeutic modalities based on tumor-specific immune reactions (5,19) and AFTV presents as a novel, stable and clinically durable vaccine that is simple to produce. In comparison with other novel and promising types of peptide vaccines, such as the Wilms tumor 1 protein vaccine, the use of AFTV does not require a preselection of patients according to the expression of tumor-associated antigens (19). The novel AFTV therapy was applied to the current study with the prospect of an additional antitumor effect. Although it was not possible to specifically measure the individual contribution of AFTV to the patient’s response, no adverse events were attributed to this treatment and the patient continues to do well.

In the present study, the highly conformal and accurate CyberKnife radiotherapy was administered to the patient in fractions. Although numerous studies have reported the use of Gamma Knife radiotherapy for GB, this approach requires the localization and immobilization of the target with the attachment of a head frame to the skull, as well as local anesthesia and the piercing of the scalp with four screws to secure the frame to the outer table of the skull. By contrast, CyberKnife radiotherapy does not require cranial tracking, as it uses the skeletal anatomy to position the radiation beam and is as precise as frame-based approaches. Furthermore, by rendering the invasive head frames unnecessary, the CyberKnife approach facilitates fractionated treatment while maintaining radiosurgical accuracy (24).

However, reirradiation of the lesion considered to be recurrent in the current patient using the CyberKnife approach was found to only aggravate the lesion. Although, further investigation using MET PET determined the lesion to be radiation necrosis rather than a recurrence. Various approaches have been reported for the treatment of radiation necrosis, including corticosteroids and surgical resection. As radiation necrosis also includes damage to the vascular endothelial cells and increases in vascular permeability, the VEGF ligand has been implicated in the pathogenesis of radiation necrosis, due to its function as a vascular permeability factor. In addition, antiangiogenic therapy with bevacizumab, which binds circulating VEGF, has been described as an effective treatment option for radiation injury (12–15).

At present, GB remains incurable and its median survival time following diagnosis is approximately one year. In addition, the ~3–5% of patients who survive for more than three years are classified as long-term survivors (25). In the present case, the pathological and clinical characteristics were highly aggressive upon the initial diagnosis. However, by utilizing a multidisciplinary treatment strategy, a successful clinical course has been achieved for three years following the initial diagnosis.
